# Panorametry: suggestion of a method for mandibular measurements on panoramic radiographs

**DOI:** 10.1186/1746-160X-5-19

**Published:** 2009-10-23

**Authors:** Edela Puricelli

**Affiliations:** 1 Oral and Maxillofacial Surgery Unit, Hospital de Clinicas de Porto Alegre, School of Dentistry, UFRGS, Porto Alegre, RS, Brazil

## Abstract

**Background:**

Orthopantomography (panoramic radiography) has been used for the study of measurements involving particularly the prediction of the eruption of impacted lower third molars and analyses of measurements of the ramus and head of mandible. The discrepancies involved with the projection of this radiographic image has stimulated the search for further ways to use it, particularly in orthodontic treatments and oral and maxillofacial surgeries. The author proposes a graphimetric method for the mandible, based on panoramic radiography. The results are expressed in linear and angular measurements, aiming at bilateral comparisons as well as the determination of the proportion of skeletal and dental structures, individually and among themselves as a whole. The method has been named Panorametry, and allows measurement of the mandible (Mandibular Panorametry) or the posterior mandibular teeth (Dental Panorametry). When combining mandible and maxilla, it should be referred to as Total Panorametry. It may also be used, in the future, with Cone Beam computed tomography (CT) images, and in this case it may be mentioned as CT Panorametry.

## Background

Panoramic radiography (orthopantomography), which technically results from collecting images with a rotating system, allows wide view of the oral maxillofacial complex, in occlusion as well as in frontal overbite. In Orthodontics and in Oral Maxillofacial Surgery, panoramic X-ray remains an important source of information. Even presenting very little image superposition, particularly for the mandible, it is not employed in comparative studies such as frontal and lateral cephalometries. As a frontolateral panoramic image of the face, it results in some distortion which involves, in decreasing degree of importance, horizontal, vertical and angular projections, respectively.

Correction of this distortion, considered the main limitation of panoramic radiography or orthopantomography, has been subject to much research since the first suggestions of technical use of the method were made. A great number of studies have explored this topic [1-11].

Graphimetric methods have been proposed by different authors in studies involving dry skulls, mandible, dental models and patients [1,3,4,8,12-25]. The distortions resulting from this type of radiograph cannot, presently, be eliminated or minimized by any type of equipment or technique. They remain, however, within acceptable proportionality, particularly for the mandible. The comparison of measurements in dental models and in panoramic X-ray, for instance, has been providing information for the establishment of methods for comparative proportion studies [3,5,6]. Based on an earlier proposal (Puricelli, 2004) [23], the author presents a graphic tracing method for the mandible, base on panoramic radiography, allowing the comparison of proportions between skeletal and dental structures, individually and as a whole. The method can also provide bilateral information, as well as establish bilateral comparison. It has been named Panorametry, and presents the possibility to measure the mandible (Mandibular Panorametry) and the posterior mandibular teeth (Dental Mandibular Panorametry). The designation Total Panorametry might be reserved for the combined analysis of mandible and maxilla. When used with computed tomography images, such as in cone-bean computed tomography, it should be mentioned as CT Panorametry.

## Methods

A viewbox, acetate matte tracing paper (0.003 inches thick, 8 × 10 inches), a compass with black lead and lead pencils in different colors, such as red, blue, yellow and green, are recommended for the task of graphic tracing. A 30-cm ruler and straightedge should also be available.

Start by placing the radiography on the viewbox. Next, place the matte acetate film over the Rx and tape it securely. Records for each graphimetric study should be identified with name, gender and date of birth of the patient, date the radiography was taken, date the study was performed, name of the professional responsible for it, reasons for which the examination is indicated, etc.

### Skeletal tracing

The sequence of steps involved in skeletal tracing is depicted below. Acronyms for the different lines and planes are presented, and in the remaining of the text they are used preceded by r or l for the right or left side, respectively.

- Structural Drawing of the Mandible

- Horizontal Reference Plane (HRP) (cartesian)

- Vertical Reference Plane (VRP) (cartesian)

- Bisector of Horizontal and Vertical Reference Planes - Line 1 (cartesian)

- Bisector Point (BP)

- Condylar Point (CP)

- Horizontal Line (rCP-lCP)

- Median Line of the Mandible (ML)

- Condilar Medial Line (CML)

- Dental Median Line (DML)

- Mental Foramen (MF)

- Line 2 (CP-MF)

- Median Point on the Gonial Area (MPGo)

- Line 3 (MF-MPGo)

- Line 4 (MPGo-CP)

- Ramus/Body Triangle I (RBT I)

- Tangents (T1-T2)

- Gonial Angle (GoA)

- Gonial Point (GoP)

- Ramus/Body Triangle II (RBT II)

- Ramus/Body Triangle III (RBT III)

- Ramus/Body Triangle IV (RBT IV)

- Ramus/Body Triangle V (RBT V)

### Dental tracing

The sequence of steps of the dental tracing includes:

- Structural Drawing of Inferior Molar Teeth

- Fixing of the Most External Points on the tooth crown Equator; and

- Tracing of the Long Axis of each tooth crown structure.

- Angle α: formed by the intersection of the long axis of the first and second mandibular molar crowns.

- Angle β: formed by the intersection of the long axis of the first and third mandibular molar crowns.

- Angle γ: formed by the intersection of the long axis of the second and third mandibular molar crowns.

To better represent and visualize the progressive tracing process, a suggestion for panorametry with colors is presented below. The multichromatic practical study of this graphimetry is individual.

**Figure 1:**
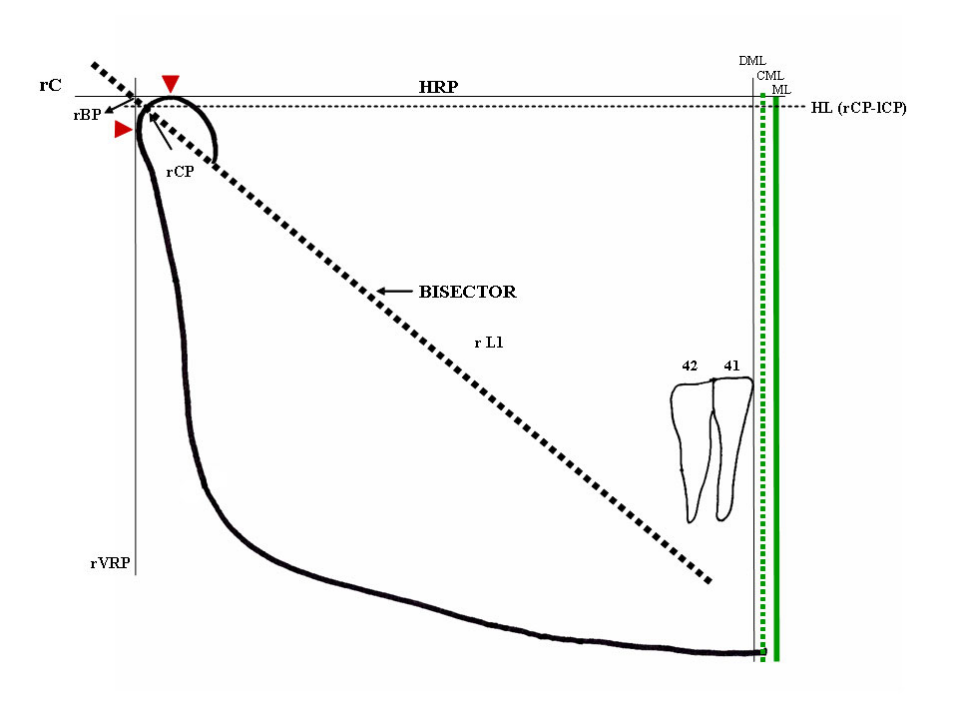
**Demarcation of the bisector of angle rC/lC and rC/VR, called (r/l)L1**. The condylar point [(r/l)CP] results from the intersection of the bisector with the anatomical tracing of the condyle. Intersection of the bisector with the HRP and VRP originates the bisector point (BP). The signal (red arrow) represents the most superior and posterior points of the external condylar surface that originate HRP and VRP. The median line (ML) (continuous green line) results from the mediatrix of the horizontal reference plane, from rBP-lBP. A Condilar Median Line (CML) (dotted green line) and a Dental Median Line (DML) (continuous black line) are also observed.

The anatomical tracing of the mandible is done with black graphite, from one condyle to the other, resulting in the structural outlining of the mandible. The most external and superior point of the condyle must then be identified on the outlines of each condyle (red arrow) (Figure 1). A horizontal line (black) named Horizontal Reference Plane (HRP), joining these points, is drawn with the ruler. The exercise will continue on the right side of the radiograph, for a better description of the method. The same procedure shoud be applied to the left side, so that the study can be completed.

The most external and posterior point of the right condyle outline is determined. A line angled at 90° with the HRP, tangent to the condyle and contacting this point, is drawn with the straightedge, and the Vertical Reference Plane (VRP), is thus obtained (black) (Figure 1). A bisector, from the intersection of HRP and VRP, is drawn with help of the compass. The resulting line, right line one (rL1), projects over the ramus and part of the mandibular body. The intersection between rL1 and the anatomical outline of the condyle is the right condyle point (rCP), and in the apex of the angle represents the bisector point (rBP) (dashed black line). Cartesian lines are thus obtained, as the geometrical principle of this study (Figure 1).

The Geometric Median Line (ML) results from drawing the HRP mediatrix from rBP and lBP (continuous green line), which can be more accurately done with use of a compass. A Condilar Median Line (CML) may be traced from rCP-lCP over the same referential horizontal plane or over the Horizontal Line (rCp-lCp). More metrical data can thus be produced (dashed green line). The Dental Median Line (DML), perpendicular to the Horizontal Reference Plane (HRP), is obtained from the inferior central interincisive point (countinuous black line) (Figure 1).

**Figure 2:**
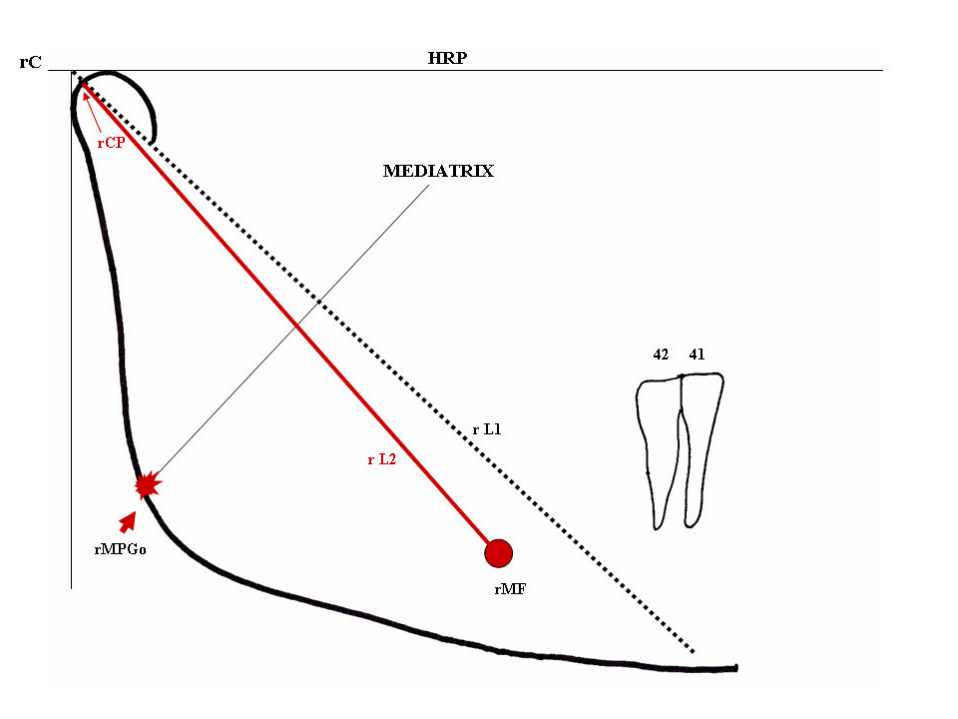
**Determination of rMGoP (arrow) from the L2 mediatrix (rCp-rMF)**.

The red graphite is then used for identification and drawing of the right Mental Foramen (rMF), with free identification and marking of its central point. A line joining the rCP and rMF points, resulting in right line 2 (rL2), is drawn (Figure 2).

From rL2, the Median Point of the Gonial Area (rMPGo) is drawn on the gonial region. Positioning the sharp point of the compass on the rMF central point, an intersection over rL2 is marked. The process is repeated from the rCP. The compass should be opened more widely than half the length of this line, for both drawings. The two intersections define the rL2 mediatrix. Its lower extension originates a new rMPGo on the external outline of the mandibular angle region. In this case, only the intersection point on the mandibular border should be marked (red) (Figure 2).

**Figure 3:**
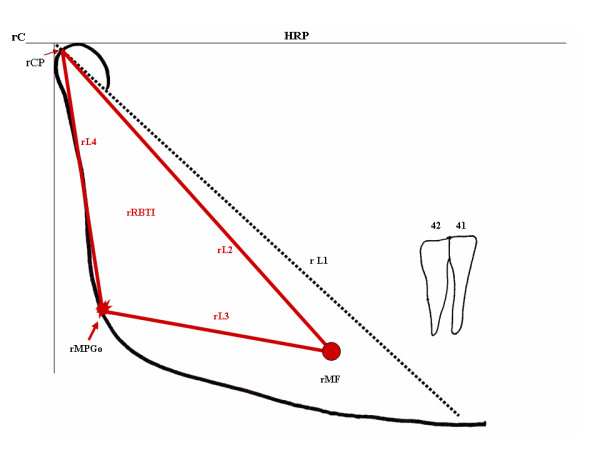
**The intersection of points rCP-rMP-rMGoP or lines L2-L3-L4 determines the rRBT I triangle**.

The right line 3 (rL3) and right line 4 (rL4) originate from joining, respectively, the rMF with the rMPGo and the rMPGo with the rCP. This results in the triangle rCP-rMF-rMPGo, or rRBT I (right Ramus/Body Triangle I), related to the skeletal structure. The triangle, in red, allows angular, linear measurements, such as for instance length of the rCP-rMF line (rL2), or rCP-rMF-rMPGo angle, which can be used in comparative studies (Figure 3).

**Figure 4:**
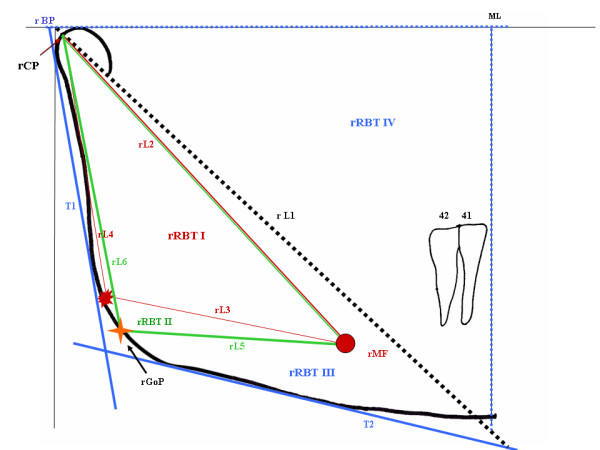
**Determination of tangents (T1 and T2) (blue) and rGoP (arrow) which will give origin to Triangles rBT II (green), rBT III (blue) and rBT IV (dotted blue)**.

From the structural outline of the mandible, two tangent lines can be traced (in blue) through the most dorsal points on the posterior surface of the condyle and ramus (rT1) and borders of the most inferior outline of the body and the region of the mandibular angle (rT2). The bisectrix of rT1 and rT2 (Figure 4) determines the right Gonial Point (rGoP) (yellow).

Another right Ramus/Body Triangle (rRBT II) can be drawn by connecting the points rCP-rMF-rGoP-rCP, allowing for the same angular and linear measurements described for rRBT I, which may be traced in green. The rRBT II is thus formed by lines L2 (red), L5 and L6 (green) (Figure 4). A third triangle rRBT III may then originate from linking tangents 1 and 2 (blue) with the right line 1 (rL1), representing a new opportunity for measurement. Its limits extrapolate the mandibular tracing (Figure 4). Similarly, the intersection of lines ML with HPR (dotted blue lines) and rL1 (dotted black line) results in a right triangle rRBT IV (Figure 4).

**Figure 5:**
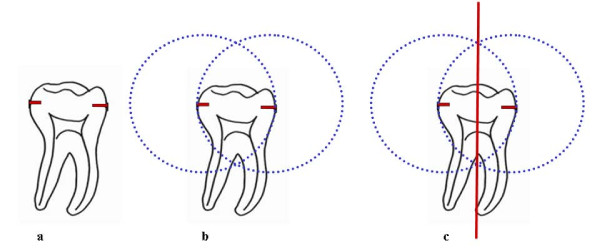
**Demarcation of the long axis of the dental crown (AX) of 46**. (a) location of points on the crown equator (red); (b) tracing with compass to position the intersection of the lines; (c) drawing of a line segment to establish the long axis of the dental crown.

The potential of the proposed protocol for the definition of tracing for dental structures may also be explored. The crown-root structures of the lower molars are drawn with black graphite, and the points corresponding to the widest region of the crown (equator), in mesio-distal orientation, are marked (red) (Figure 5a).

**Figure 6:**
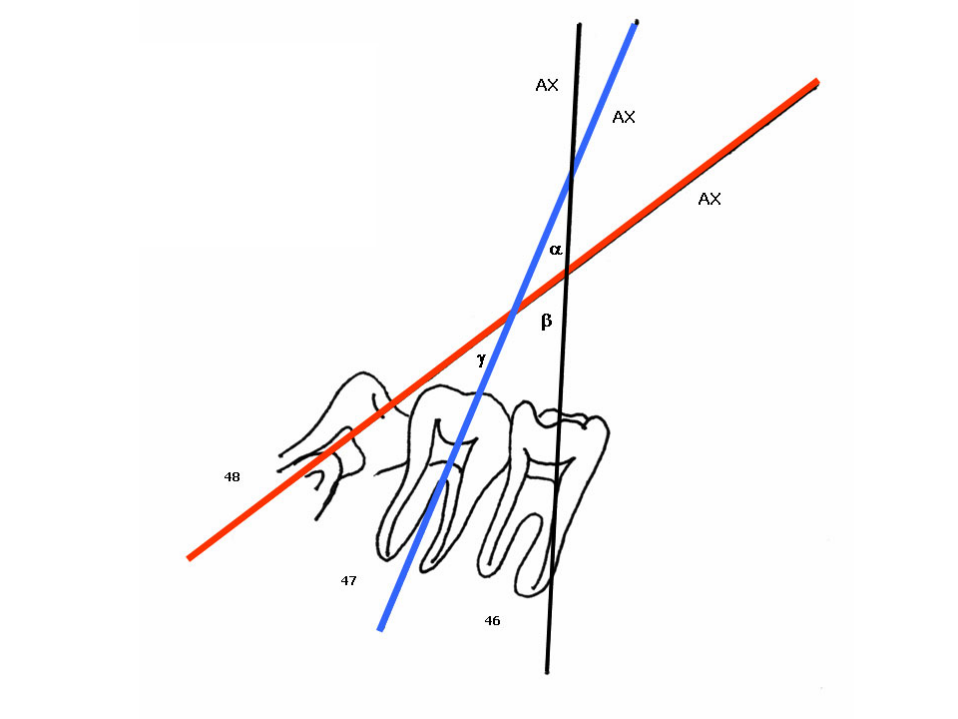
**Determination of the interdental angular dimensions: αr, βr and γr**. 46AX-47AX = Aαr 46AX-48AX = Aβr 47AX-48AX = Aγr

The sharp point of the compass is placed on the mesial point of the first molar crown and the graphite is placed on the distal outline of the same crown, overlaying the tracing on this point. A semicircle is then drawn in cervical directed to the occlusal, extending transversally to the tooth long axis. After repositioning the sharp tip of the compass on the distal point, and tracing the external border of the same tooth, the semicircle is repeated (Figure 5b). As a result, two intersections, one cervical and the other occlusal, determine the long crown axis (AX) (Figure 5c). The intersection of these paired lines determines the r Aα, r Aβ and r Aγ angles (Figure 6).

**Figure 7:**
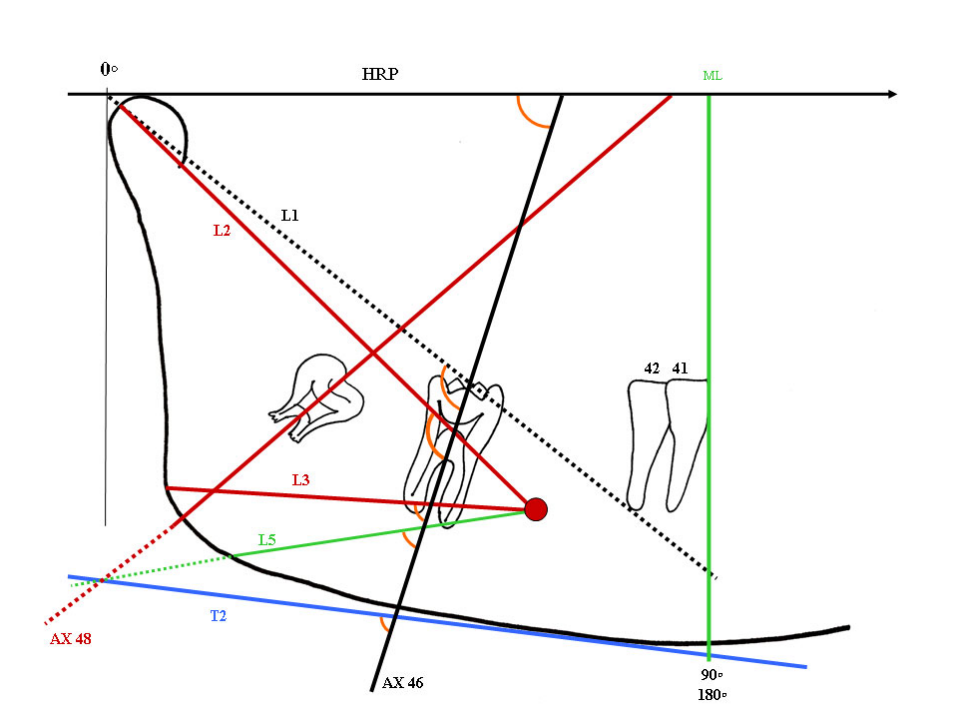
**As an example, the orientation for determination of the different angles resulting from intersections of the long crown axis of the 46 with the HRP (0 to 90 degrees) and lines L1 L2;L3;L5 and T2 (0 to 180 degrees) is presented**. The angles are shown in orange. Measurements extending to the mandibular dimensions are also recorded (such as T2;L5 and 48 AX lines).

The two intersection points of the first molar crown drawing are joined using the black graphite. The line is extended to the Horizontal Plane Reference, through rL1, and continues downwards through rL2, rL3 and rL5R, reaching rT2. The process is repeated for the second and third molars, for which blue and red graphite, respectively, are recommended. The intersection of each of the long crown axis, when crossing lines rC-lC (HRP), Horizontal Line(HL) rL1, rL2;rL3;rL5 and rT2, originates new angles which can be used for sequential measurements (Figure 7).

**Figure 8:**
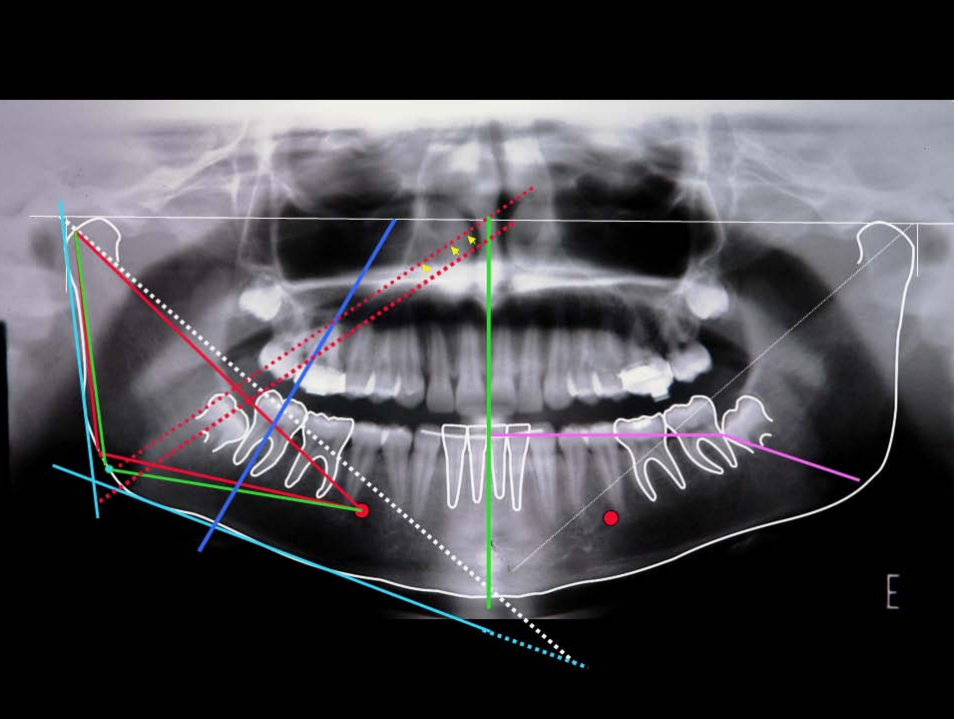
**Image of panorametry traced over a panoramic radiography with information for bilateral bone-dental angular measurements of the mandible**. For clarity, the different possibilities described in the text are not presented (they may be seen in the preceding figures). Besides different linear or angular measurements, the triangular areas may also be measured and compared in terms of surface. See adequacy of 48 AX (arrows) for determination of its angle with HRP. We also point out the possibility of linear intercoronaly measurements, such as M 38- ML (90°) and M 38- l MPGo (pink).

An example of the image of this graphimetry, over a panoramic radiograpy resulting from overlaying the images described, shows many of the measurements which can be applied to the inferior jaw (Figure 8). Linear measurements, correlating the molars among themselves or with MPGo, ML (pink), CML and DML among others, may be suggested from the points marked on the dental crowns.

**Figure 9:**
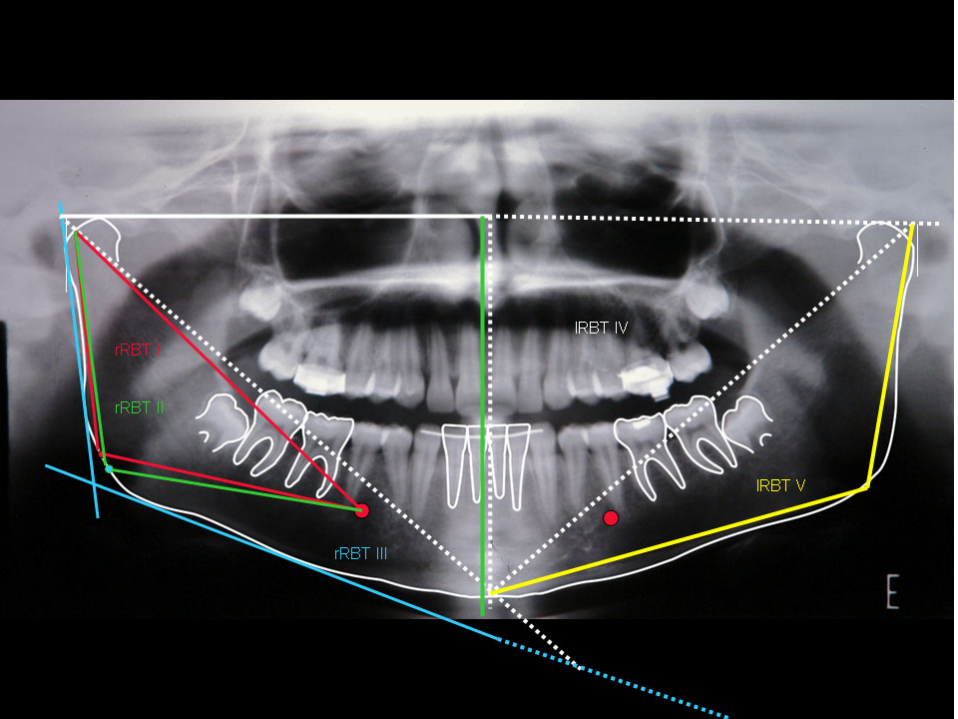
**We point out the measurements within the area of the mandibular body and ramus, rRBT I (red) and rRBT II (green)**. Triangles rRBT III (blue) l RBT IV (white) and lRBT V (yellow) go beyond mandibular body and ramus measurements. The common intersection point for these triangles is in the BPs.

In sequence, different possibilities of triangle tracing are presented. The design of a triangle l RBT V from l BP, l MPGo and LM is stressed. A triangle RBT VI may also be created from these points, just by replacing MPGo by GoP (Figure 9).

## Results

This graphimetric study proposes linear (vertical and horizontal) and angular measurements of mandibular and dental structures, individually, as a whole and bilaterally, in the same radiography. It may also allow other studies, such as comparative longitudinal measurements. The rBP and lBP points are considered as zero degrees (0°). The intersection between ML and the inferior border of the chin determines 90° for both the right and the left sides. For CML and DML, the same angular gradation is applied. The projections of the long crown axis (AX) on the HRP or this plane (rC-lC) on the Horizontal Line (HL) are scored from 0 to 90 (degrees). The lines may go beyond ML, CML and DML, particularly in case of acute angles of the third molar, and their transfer to a parallel line (Figure 8, tooth 38 arrows) may then allow measurement. On the other hand, the angles of the different intersections (AX) can be progressively measured on the lines that follow (L1, L2, L3 and L5 and T2). In this case, they may also be measured from 0 degree in each side, right or left, and may reach up to 180 degrees (Figure 7).

For linear measurement of rT1, its intersection with the Horizontal Reference Plane and with rT2 are considered as the farthest borders. The second tangent line, rT2, is distally limited by the rT1 intersection, whereas its mesial border lies at the intersection with ML extension (Figure 4). In case of interest on the metric relationship with CML or DML the procedure is repeated.

The linear measurements related to ML, CML and DML should proceed from a right angle with the geometric referential, the median lines in this case. Internal and external angles contained in the different triangles are evaluated according to trigonometric patterns. All sides of each triangle, medians and bisectors may also be measured and recorded for statistical and comparative studies. The Aα, Aβ and Aγ angles allow a well focused study of crown angulation among themselves.

Since dental crown landmarks are clearly visible, interdental measurements may also be suggested. Image distortions seem to be smaller in this area. For official recording of the measurements, their standardization is suggested as presented in Table 1, Table 2, Table 3, Table 4, Table 5, Table 6, Table 7 and Table 8.

**Table 1:** Bone transversal linear dimensions.

**Tracing**	**Length (mm)**
rBP-lBP	
rCP-lCP	
rMF-lMF	
rMPGo-lMPGo	
rGOP-lGOP	

**Table 2:** Bone unilateral linear dimensions.

Tracing*	**Length (mm)**
rBP-ML	
rBP-CML	
rBP-DML	
rCP-ML	
rCP - CML	
rCP-DML	
rMPGo-ML	
rMP- CMP	
rMPGo-DML	
rGoP - ML	
rGoP-CML	
rGoP -DML	
rMF-ML	
rMF-CML	
rMF-DML	

* The measurements should be obtained from a 90° angle with lines ML, CML and DML.

**Table 3:** Bone linear dimensions.

**Tracing**	**Length (mm)**
rCP-rMF = L2	
rMF-rMPGo = L3	
rMPGo-rCP = L4	
rMF-rGoP = L5	
rGoP-rCP = L6	
rT 1	
rT 2	
rL1-ML (r CP -ML)	
rL1-CML(r CP- CML)	
rL1-DML (r CP -DML)	

**Table 4:** Bone angular dimensions.

**Angle**	**Degrees**
lBP-rBP-rMPGo	
lBP-rBP-rGoP	
lBP-rBP-rMF	
lCP-rCP-rMPGo	
lCP-rCP-rGOP	
lCP-rCP-rMF	
rT 1 -rT 2	
rL1-ML	
rL1-CML	

**Table 5:** Angular ramus/body triangular dimensions.

**Angle**	**Degrees**
rL2-rL3	
rL3-rL4	
rL2-rL4	
rL2-rL5	
rL5-rL6	
rL2-rL6	
rT 1-rT 2	
rT 1 - rL1	
rT 2-rL1	
rL1-ML	
ML-rBp (HPR)	
ML-rBP-L1	

This example is not limited to the median line, and may be repeated with CML and ML.

**Table 6:** Intercoronary linear dimensions.

**Tracing**	**Values (mm)**	**Tracing**	**Values (mm)**
M46-rMPGo		M36-lMPGo	
M46-rGoP		M36-lGoP	
M46-D48		M36-D38	
M46-D47		M36-D37	
M46-ML (90°)		M36-ML (90°)	
M46-CML (90°) M46-DML(90)		M36-CML (90°) M36-DML(90)	

This table represents an example of measurements that may be individually repeated for each tooth, from its mesial (M) and distal (D) crown aspect. Measurements should be done at the crown equator.

**Table 7:** Dento-mandibular angular dimensions.

**Angle**	**Degrees**
46AX-rBP/lBP (HRP)	
46 AX- rCP-l CP (HL)	
46AX-rL1	
46AX-rL2	
46AX-rL3	
46AX-rL5	
46AX-rT 2	

The angle results from the intersection of its long axis of the dental crown (AX) with the different lines (the procedure is repeated for all inferior molars in both sides).

**Table 8:** Interdental angular dimensions.

**Angle**	**Degrees**	**Angle**	**Degrees**
Aα 46-47		Aα 36-37	
Aβ 46-48		Aβ 36-38	
Aγ 47-48		Aγ 37-38	

## Discussion

Skull lateral teleradiographs are widely accepted for cephalometric studies, due to the amount of information about measurement of dental and craniofacial complexes they provide. However, the overlaying of anatomical structures makes the identification of marker points more difficult and prevents comparisons between the left and right sides [4,14]. The comparison of cephalometric and craniometric measurement in lateral teleradiographs has shown that the method has little reliability in the evaluation of the gonial angle, with a distortion which is in average larger for the gonial angle closer to the film [5]. Even allowing for latero-lateral studies, postero-anterior face images in cephalostat present marker anatomical points of difficult definition.

The method of panorametry proposed here allows the recognition, from horizontal and vertical reference planes, of a skeletal and a dental median lines, in a mandible analyzed independent of the rigid structures of the facial skeleton [23]. Contrary to the proposal made by Larheim and Svanaes (1986) [8], in our experience some asymmetry may exist between the skeletal structure and the dental arch without necessary classification of the Median Line (ML) as only one. The possibility to determine a Condilar Median Line (CML), established from the distance between right and left Condylar Points (rCP-lCP), may also be used for these measurements. Determination of the Median Line from rBP-lBP follows, therefore, the Cartesian principle that from three coordinates - reference horizontal and vertical planes and their bisector - the mandibular structure can be viewed spatially. The Dental Median Line (DML), however, is directly related to the symmetry of the lower dental arch. These median lines may occasionally superimpose, which could indicate a better relation of the bone-dental symmetry.

The Mental Foramen (MF) stands out as an anatomical point, but may be difficult to locate due to lack of uniformity of its border. It may be delineated, however, by observation of the path of the nervous conduct between the pre-molar apices, under adequate light.

The Median Point of the Gonial Area (MPGo) in our methodological proposal starts at line L2(r/l), and is not meant to be defined as the angle referred to in former studies. It is geometrically determined, and is part of the triangular figure formed from points (r/l)CP - (r/l)MF - (r/l)MPGo or (r/l)L2 - (r/l)L3 - (r/l)L4.

The presence of lines tangent to mandibular ramus (T1) and body (T2) reproduces the proposal by Mattila et al. (1977) [4] for determination of the gonial angle in orthopantomograms. Subsequent studies showed that the gonial angle is more accurate, more stable and presents less distortions, even with variation in the position of the patient head [4,7-11]. In our experience, the bisector of the intersection of the tangent lines allows the determination of the Gonion Point (GoP), as a further referential information to investigate.

Determination of the Gonion Point (GoP)allows the outline of a further triangle, also called Ramus/Body Triangle II, using points (r/l)CP - (r/l)MF - (r/l)GoP). This triangle, as with RBT I, is also contained within the limits of the mandibular area. The RBT III triangle [(r/l)T1 - (r/l)T2 - (r/l)L1] can associate external and internal metrics of the mandibular body and ramus.. Intersection of line (r/l)ML-L1 and the Horizontal Reference Plane originates a right triangle, which creates through its bisector a new Cartesian reference near the mandibular gonial region. New triangles are thus determined, from pre-existing and well established points. Presenting its own angular and linear dimensions, this triangle allows unilateral and bilateral comparison to be performed, in longitudinal studies as well. This ample graphimetric view show the mandibular ramus and body still in triangular shape, allowing for surface studies. We may suggest that, based on the presente proposal in which the cartesian tracing evolves from the condyles, the tracing for Condylar Morphology Scale (CMS) and ramus height analysis mentioned by Borstlap et al. (2004) [22], and the asymmetry indices according to Habets et al (1988) [15] and Kjellberg et al. (1994) [17], are reintroduced for new studies.

Using panoramic radiography, among other measurements it is possible to observe molar spaces in the same film and associate the eruptive process of third molars with other dento-facial structures [1-3,6,13,14,16,18,20,21,23-25].

Horizontal measurements are considered less accurate [8,9]. Linear intercrown measurements proposed in the present work allow the observation of possible combinations of the first molar with the second and third molars, and of these two among themselves, uni- or bilaterally. It also allows extension of measurements for ML, CML and DML in a 90 degree relationship Welander et al. (1989) [9] suggested that the anterior mandibular area is more susceptible to distortions. The present study suggests a more reliable method, based on the establishment of a geometric relationship of ML, CML and DML in right angle with the Horizontal Reference Plane, where lines rBP/lBP and rCP/lCP may also be used.

Angular measurements of the teeth performed with panoramic radiography are more reliable, when compared to other radiographic methods [3,10,12,13]. Angular distortions and variability are more frequently concentrated in the pre-molar region and canines of both arches, whereas the molar region and the inferior borders of the mandibular body and posterior border of the ramus are relatively stable. Measurement of the gonial angle was also remarkably reproducible, even with largely different types of head positioning. Angular measurements resulting from panoramic radiographs are thus perfectly adequate for quantitative studies, particularly of the development of posterior dental regions and of inferior third molars [7].

In studies reported by Altonen et al. (1977) [12] and Hattab et al. (1999) [20], the longitudinal axes of these teeth were drawn through the midpoint of the occlusal surface and bifurcation or the midpoint of the bone concentration forming this bifurcation. Catella et al. (1998) [19], on the other hand, projected the long axis of the teeth by a line bisecting the midpoints between the mesial and distal height of contours and the cementoenamel junction. If the cementoenamel junction had not already formed, the long axis was determined by a line perpendicular to the line connecting the mesial and distal heights of contour on the developing crown. In the search for a more geometrical form, of better visual and graphic identification, we propose drawing the long axis of the tooth based on the crown structure. This design, however, allows the drawing of images with deviations between the root and the crown of the same tooth, so that the correct long axis proposed is not adequately followed. Precision in the dental outlines, as provided by different methods, is one of most important factors contributing for reliability and reproducibility of graphimetric results.

We do not propose a comparison of the β angle introduced by Altonen et al. (1977) [12] with the γ angle presented here, since they are obtained with different approaches. Our concern with increasing the options of angular measurements is based on the results of Frykholm et al. (1977) [3]. The reliability of panoramic radiography in providing angular measurements of adjacent teeth was evaluated, and the authors concluded that this is the most adequate radiographic method for the analysis of dental angulation. The studies by Zach et al. (1969) [1] have also prompted us to explore the possibility of using this type of radiographic image for longitudinal investigations, following for instance the growth of a child and predicting any possible impaction due to due to lack of space.

In our first methodological proposal (Puricelli, 2004) [23], graphimetric data were enhanced, including linear and angular measurements for comparative studies of mandibular and dental structures. New graphic inclusions and metric proposals are introduced in the present work.

## Conclusion

Considering that currently there is a lack of methodological approaches to explore results from panoramic radiography, this work proposes a standardizing method for the establishment and performance of skeletal and dental measurements of the mandible.

The reference points suggested are predominantly located in the region between the molars and mandibular ramus, for which image distortions are known to be smaller. The suggested tracing method meets the needs of skeletal and dental measurements, uni- and/or bilaterally. Measurement of triangular surfaces may be explored in a future study. The apparent excess of information generated intends to allow maximal levels of comparison, indicating the measurements more suitable and reliable for each situation, without the intention to exhaust all possibilities. Statistical studies with high degree of confidence will certainly allow the indication of the most recommended measurements. Currently, this proposal is not limited to radiographs but contemplates also the possibility to study panoramic CT images, particularly those obtained with Cone Beam CT.

## Skeletal abbreviations

(preceded by r or l, for right or left sides)

BP: Bisector Point; C: condyle; CML: Condilar Median Line; CP: Condylar Point; DML: Dental Median Line; GoA: Gonial Angle; GoP: Gonion Point; HL: Horizontal Line; HRP: Horizontal Reference Plane; L1 to L6: lines 1 to 6; MF: Mental Foramen; ML: Median Line of the Mandible; MPGo: median point of the Gonial Area; RBT I: Ramus/Body Triangle I; RBT II: Ramus/Body Triangle II; RBT III: Ramus/Body Triangle III; RBT IV: Ramus/Body Triangle IV; RBT V: Ramus/Body Triangle V; T: Tangents; VRP: Vertical Reference Plane

Dental abbreviations

A α: alpha angle; A β: beta angle; A γ: gamma angle; AX: Long Crown Axis

## Competing interests

The author declares that they have no competing interests.
